# The Impact of Surgery and Radiotherapy on Health-Related Quality of Life of Individuals with Oral and Oropharyngeal Carcinoma and Short-Term Follow up after Treatment

**DOI:** 10.31557/APJCP.2020.21.5.1227

**Published:** 2020-05

**Authors:** Marcelo Coelho Goiato, Andressa Paschoal Amoroso, Bruna Silva, Emerson Gomes dos Santos, Fernanda Pereira de Caxias, Sandro Basso Bitencourt, Amália Moreno, Daniela Micheline dos Santos

**Affiliations:** 1 *Oral Oncology Center and Department of Dental Materials and Prosthodontics, São Paulo State University (UNESP), School of Dentistry, Araçatuba, SP, Brazil. *; 2 *Department of Business, Paulista School of Politics, Economics and Business, Universidade Federal de São Paulo (UNIFESP), Osasco, Brazil. *; 3 *Department of Oral Surgery, Pathology and Clinical Dentistry, School of Dentistry, Universidade Federal de Minas Gerais (UFMG), Belo Horizonte, Brazil. *

**Keywords:** Mouth neoplasms, oropharyngeal neoplasms, quality of life, surgical oncology, radiotherapy

## Abstract

**Objective:**

This study aimed to evaluate the quality of life of patients with oral or oropharyngeal cancer by using specific questionnaires (QLQ-C30 and QLQ-HandN35), varying according to the location of the tumor (oral cavity or oropharynx) and the treatment performed (only surgery or surgery associated with radiotherapy).

**Methods:**

Fifty patients were enrolled in this study and answered the EORTC QLQ-C30 and EORTC HandN35 questionnaires, before (baseline), at 1 week, and 3 months after treatment. Internal consistency reliability was calculated with the Cronbach coefficient. The Kruskal-Wallis and Wilcoxon tests were applied and P<.05 was considered significant.

**Results:**

Some aspects showed significant difference (P<.05) between tumor location (oral/oropharyngeal) and treatment performed (surgery or surgery plus radiotherapy) only for the baseline and 1 week after treatment periods.

**Conclusion:**

Quality of life is a factor that affected patients with head and neck cancer. The time elapsed after treatment, whether surgical or surgical plus radiotherapy, influenced patient quality of life. The period of greatest morbidity was 1 week after treatment.

## Introduction

Head and neck carcinoma is a group of neoplasms that can be located in several areas, such as the lip, oral cavity, tonsils, oropharynx, hypopharynx, nose, as well as the paranasal sinus, larynx, parotid, and thyroid (Shavi et al., 2015; Licitra et al., 2016; Nyqvist et al., 2016). 

The squamous cell carcinoma (SCC) is the most common type of oral carcinoma (Gregorie, et al., 2010)and its approach must be individual and based on staging and the curative treatment is performed by the surgical resection of the tumor. Radiotherapy also shows curative results in small lesions. Furthermore, in some cases, surgery is accompanied by post-surgical radiotherapy to consolidate the treatment (Nyqvist et al., 2016; Janssens et al., 2016). Thus, surgery and radiotherapy are the primary treatment procedures, but chemotherapy also has its place in the palliative treatment of head and neck relapsed tumors (Biordal et al., 2011; Morton, 2003).

Diagnosis and treatment of head and neck cancer have a severe impact on the quality of life of patients (Janssens et al., 2016; Rigoni et al., 2016; Ojo et al., 2012) as well as their relatives (Rigoni et al., 2016; Ojo et al., 2012). Quality of life is based on the individual experience of each person (Calver et al., 2018) and it linked to an individual’s present experience and their hopes and expectations at a particular time point (Calman, 1984). Communication capacity, ability to eat without help, aesthetics, taste, smell, and sexual life are priorities for the patient in treatment, and have an impact on their quality of life perception (Arslon et al., 2016); therefore, maintenance of these characteristics is essential to people living with cancer (Mucke et al., 2015). However, quality of life maintenance may be influenced by several factors such as age, surgical extension, toxicity, and chronic or acute side effects to the treatment in association with radiotherapy and chemotherapy, which may alter basic and vital life functions, such as breathing, feeding, and oral communication (Licitra et al., 2016, Nyqvist et al., 2016; Herrero et al., 2003). Furthermore, changes in appearance, difficulty swallowing, and pain can cause problems in social functions such as social eating, influencing the quality of life of patients with oral or oropharyngeal cancer (Williamson et al., 2011; Braz et al., 2005). Based on the recognition of the importance of quality of life, there was an expansion of evaluation criteria for these issues in these patients (So et al., 2012). 

In order to promote a standard and detailed assessment of people living with cancer, several questionnaires were proposed. Among the most used is the Questionnaire about Quality of Life (QLQ-C30) from the European Organization for Research and Treatment of Cancer (EORTC), as well as the specific module for patients with head and neck cancer (QLQ-HandN35) (Janssens et al., 2016; Aaronson, 1993). These questionnaires were created to evaluate the quality of life in clinical trials, used together with specific diagnostic tools with the purpose of enlarging the coverage, sensitivity, and specificity of the quality of life evaluation of these patients (Janssens et al., 2016; Bjordal et al., 1999).

Although quality of life is a well explored subject, the literature is still scarce in studies that compare the impact of cancer treatment for tumors located in the oral cavity and oropharynx, and for treatment by surgery or surgery associated with radiotherapy with short-term follow up. 

In view of the above, this study aims to evaluate the quality of life of patients with oral or oropharyngeal cancer by using specific questionnaires (QLQ-C30 and QLQ-H and N35), varying according to the location of the tumor (oral cavity or oropharynx) and treatment performed (surgery or surgery associated with radiotherapy). The null hypothesis tested was that there would be no difference in the pattern of patient quality of life between the different treatments and the tumors location.

## Materials and Methods

*Patients recruitment*

The participants of this study were patients from the Center of Oral Cancer of Sao Paulo State University (UNESP), Araçatuba Dental School, where the study was done. The patients were invited by phone and during appointments in the Dental School. The interviews were presential and the investigators read the questions to the patients because some of them were illiterate. 

Thus, fifty patients (9 females and 41 males) were selected according to the inclusion criteria, which were: (1) patients diagnosed with head and neck cancer (oral or oropharyngeal); (2) submitted to treatment with surgery or surgery with radiotherapy (associated with or without chemotherapy); (3) with cognitive ability, understanding, and capacity to answer the questions from the EORTC QLQ-C30 and QLQ-H and N35 questionnaires. 

The demographic data were obtained by a detailed anamnesis. The EORTC QLQ-C30 and QLQ-H and N35 questionnaires were used in three periods: at the first appointment after diagnosis (baseline), 1 week after, and 3 months after the end of treatment. The questionnaires were applied at the clinic and they were read by the researches since some of the participants were illiterate and the participants chose the answers that applied to themselves. The patients were divided according to tumor location (oral cavity or oropharynx) and according to the treatment performed (surgery or surgery associated with radiotherapy). The data were compared according to tumor location and treatment modality in the different assessment periods.

*Ethical Approval*

The experiments were undertaken with the understanding and written consent of each subject according to recommendations of the Ethics Committee of the Araçatuba Dental School- FOA/UNESP 2.116.702/2017 and according with Declaration of Helsinki. 

*Cancer diagnosis and treatments*

The diagnosis was obtained by anamnesis and clinical and complementary exams, such as image exams (radiography, ultrasonography, resonance) and laboratorial exams (punctures with histological analysis and hemograms) by oncologic doctors that decided the adequate treatment for the patients.

*EORTC QLQ questionnaires*

The quality of life assessment was performed by the application of the Brazilian-Portuguese versions of the EORTC QLQ-C30 and QLQ-H and N35 questionnaires. The EORCT QLQ-30 questionnaire has different versions, and the one used in this study (Version 3.0) is considered a standard for research (Aaronson, 1993). As well as the EORCT QLQ-30, the EORCT QLQ-H and N35 has also been used in many clinical studies (Aaronson, 1993; Jansenet al 2016; Piesker et al, 2016; Batioglu-Karaaltin et al., 2017). Among the 710 studies included in the Ojo et al., (2012) systematic review, the QLQ-H and N35 questionnaire was the most used, being present in 244 studies published in the analyzed period. Based on this, both questionnaires were used in this study.

The EORTC QLQ-30 (Version 3.0) questionnaire has 30 questions, with scores varying from 0 to 100, that are divided into global health status (QL2) measurement, five function scales: physical (PF2), psychological (RF2), cognitive (CF), emotional (EF), and social (SF), and eight symptom scales: pain (PA), nausea/vomit (NV), fatigue (FA), dyspnea (DY), insomnia (SL) appetite loss (AP), constipation (CO), diarrhea (DI), and financial difficulties (FI) scale (Aaronson, 1993). In the QLQ-C30 questionnaire, the higher the score in global health status (QL2), the better the patient quality of life is; and the higher the score on the functional scales (PF2, RF2, CF, EF and SF), the healthier that function is. However, the higher the score on the symptom scales (FA, NV, PA, DY, SL, AP, CO, DI and FI) the worse the symptoms are (Aaronsson, 1993). For its part, the EORTC QLQ-HandN35 questionnaire is used in studies comprised of individuals with head and neck cancer, and it varies in terms of modality of treatment and disease stage (Bjordal et al., 1999). This questionnaire has 35 questions divided in seventeen scales: pain (HNPA), swallowing (HNSW), sensorial problems (HNSE), speech problems (HNSP), trouble with social eating (HNSO), trouble with social contact (HNSC), sexuality (HNSX), teeth (HNTE), problems opening the mouth (HNOM), dry mouth (HNDR), viscous saliva (HNSS), coughing (HNCO), feeling ill (HNFI), pain killers (HNPK), nutritional supplements (HNNU), feeding tube (HNFE), and weight gain (HNWG) (Bjordal et al., 1999). The results are calculated by scores given to the answers (1=Not at all; 2=A little; 3=Quite a bit; 4=Very much) (variation from 1=Horrible to 7= Excellent); (1=No and 2=Yes). For all items and scales, higher scores infer more problems. The score approach for both the QLQ-HandN35 and QLQ-C30 questionnaires is identical. 

*Statistical analysis*

Descriptive statistical analyses (including frequency distributions and percentages) were performed on the patient demographic data and the responses to both questionnaires. Internal consistency reliability was calculated by using the Cronbach Alpha Coefficient (α), which identifies whether there is correlation between items, only for questions that had more than one item (Bland and Altman, 1997; Cronbach, 1951). This coefficient score varies from zero to 1; the closer to 1, the better the result. Preliminary 2 (for categorical measures), Mann-Whitney, and Kruskal-Wallis tests were used to examine group differences in demographic and medical variables. Mann-Whitney tests were used to test for differences in the EORTC scores between cancer patients and treatments, and Kruskal-Wallis analyses of variance were used to examine differences between the cancer treatment periods. All significance tests were 2-tailed and P<.05 was considered significant.

**Table 1:** Characteristics of Patients in Relation to Demographic and Clinical Variables of Interest (n=50).

Variables	Oral and oropharyngeal carcinoma ‡
Sex	
Female	9 (18.0)
Male	41 (82.0)
Age (years) *	61.5 (13.1)
Marital Status	
Single	11 (22.0)
Married	26 (52.0)
Divorced	4 (8.0)
Widower	9 (18.0)
Occupation	
Working	32 (64.0)
Retired	18 (36.0)
Diagnosis	
Squamous cell carcinoma	42 (84.0)
Other pathologies	8 (16.0)
Anatomical site	
Oral	36 (72.0)
Oropharyngeal	14 (28.0)
Treatment	
Surgical	36 (72.0)
Surgical + Radiotherapy	14 (28.0)
Habit	
≤ than 2 habits	22 (44.0)
> than 2 habits	28 (56.0)
Family history	
Yes	24 (48.0)
Do not know	26 (52.0)
Dental arch	
Dentate total	3 (6.0)
Partial edentulous	29 (58.0)
Total edentulous	18 (36.0)
Type of prosthesis	
Complete removable prosthesis	16 (32.0)
Partial fixed prosthesis	11 (22.0)
Do not use	23 (46)
Teeth removed due to treatment	
Yes	17 (34)
No	33 (66)

‡,Values in parentheses are expressed as percentage; *, Mean value (standard deviation).

**Figure 1:**
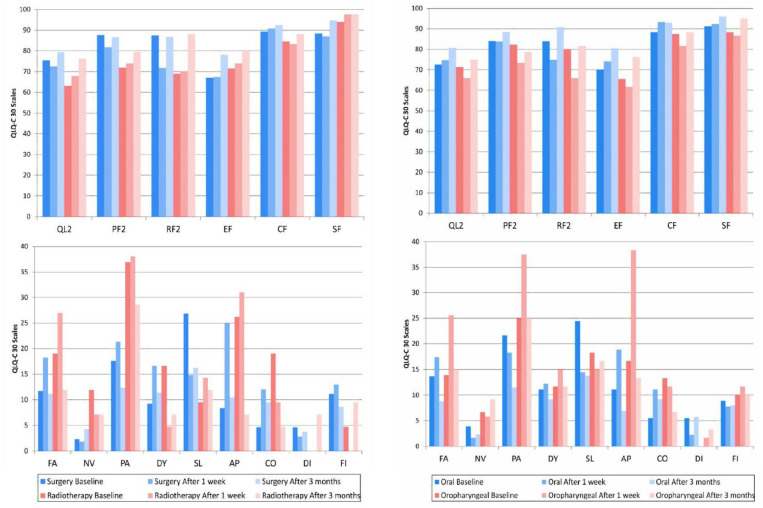
Mean Scores from European Organization for Research and Treatment of Cancer (EORTC) QLQ-30 for Patients with Head and Neck Cancer for Treatment Group Differences (Surgery only [n=36] and Surgery + Radiotherapy [n=14]); and Cancer Location Differences (Oral [n=30] and Oropharyngeal [n=20]) at Different Phases of Treatment (baseline; after 1 week, and 3 months). QL, Global quality of life; PF, Physical functioning; RF, Role functioning; EF, Emotional functioning; CF, Cognitive functioning; SF, Social functioning; FA, Fatigue; NV, Nausea/vomiting; PA, Pain; DY, Dyspnea; SL, Sleep disturbance; AP, Appetite; CO, Constipation.; DI, Diarrhea and FI, Financial difficulties. (Scales are not displayed if the means for groups equal 0).

**Table 2:** Reliability Coefficients and Descriptive Statistics for EORTC QLQ-C30 (n=50).

EORTC QLQ-C30 Scales	Baseline		After 1 week		After 3 months	
	Mean (SD)	Coefficient α	Mean (SD)	Coefficient α	Mean (SD)	Coefficient α
Global health status/QoL (QL2)	72.00 (23.62)	0.89	71.17 (18.69)	0.90	78.40 (17.67)	0.92
Physical functioning (PF2)	83.33 (23.18)	0.85	79.60 (20.18)	0.76	84.49 (15.48)	0.68
Role functioning (RF2)	82.33 (24.84)	0.85	71.33 (25.43)	0.74	87.07 (17.76)	0.78
Cognitive functioning (CF)	68.17 (29.87)	0.86	69.17 (20.84)	0.66	78.74 (20.13)	0.75
Emotional functioning (EF)	88.00 (16.16)	0.15	88.67 (16.30)	0.45	91.16 (16.7)	0.54
Social functioning (SF)	90.00 (20.48)	0.74	90.00 (19.92)	0.83	95.58 (8.86)	0.34
Fatigue (FA)	13.78 (22.40)	0.83	20.67 (20.45)	0.73	11.34 (17.20)	0.74
Nausea/vomiting (NV)	5.00 (13.57)	0.74	3.33 (12.14)	0.57	5.1 (12.36)	0.53
Pain (PA)	23.00 (25.40)	0.62	26.00 (24.08)	0.84	17.01 (21.11)	0.69
Dyspnea (DY)	11.33 (20.88)	-	13.33 (20.20)	-	10.20 (18.26)	-
Insomnia (SL)	22.00 (32.72)	-	14.67 (20.38)	-	14.97 (19.32)	-
Appetite loss (AP)	13.33 (25.20)	-	26.67 (27.77)	-	9.52 (16.67)	-
Constipation (CO)	8.67 (21.09)	-	11.13 (22.95)	-	8.16 (19.87)	-
Diarrhea (DI)	3.33 (10.10)	-	2.00 (8.00)	-	4.76 (11.79)	-
Financial difficulties (FI)	9.33 (20.25)	-	9.33 (23.27)	-	8.84 (16.35)	-

EORTC indicates European Organization for Research and Treatment of Cancer; ellipses, no coefficient α calculated (single-item scale). Higher scores reflect better functioning for “Physical functioning” through “Global quality of life” scales; Lower scores reflect better functioning for “Fatigue” through “Financial difficulties” scales.

**Figure 2:**
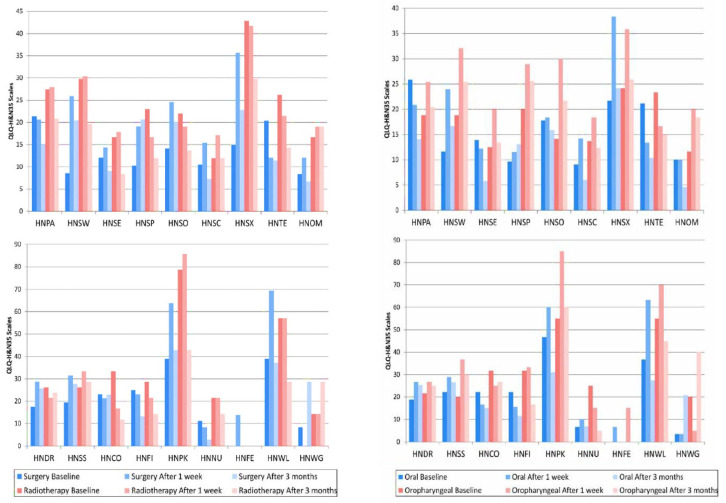
Mean Scores from European Organization for Research and Treatment of Cancer (EORTC) QLQ-HandN35 for Patients with Head and Neck Cancer for Treatment Group Differences (Surgery Only [n=36] and Surgery + Radiotherapy [n=14]); and Cancer Location Differences (oral [n=30] and oropharyngeal [n=20]) at Different Phases of Treatment (baseline; after 1 week, and 3 months). HNPA, Pain; HNSW, Swallowing; HNSE, Senses; HNSP, Speech; HNSO, Social eating; HNSC, Social contact; HNSX, Sexuality; HNTE, Teeth; HNOM, Problems opening mouth; HNDR, Dry mouth; NHSS, Sticky saliva; NHCO, Coughed; HNFI, Felt ill; NHPK, Painkillers; HNNU, Nutritional supplements; HNFE, Feeding tubes; HNWG, Weight gain, and HNWG, Weight gain. (Scales are not displayed if means for groups equal 0).

**Table 3:** Reliability Coefficients and Descriptive Statistics for EORTC QLQ-HandN35 (n=50).

EORTC QLQ-HandN35 Scales	Baseline		After 1 week		After 3 months	
	Mean (SD)	Coefficient α	Mean (SD)	Coefficient α	Mean (SD)	Coefficient α
Pain (HNPA)	23.00 (22.50)	0.70	22.67 (17.98)	0.59	16.67 (16.84)	0.64
Swallowing (HNSW)	14.50 (24.56)	0.86	27.17 (23.44)	0.76	20.24 (22.76)	0.84
Senses problems (HNSE)	13.33 (24.51)	0.76	15.33 (22.55)	0.63	8.84 (15.99)	0.69
Speech problems (HNSP)	13.78 (20.64)	0.71	18.44 (23.72)	0.72	18.14 (24.08)	0.79
Trouble with social eating (HNSO)	16.33 (25.86)	0.92	23.00 (21.73)	0.81	18.20 (22.74)	0.90
Trouble with social contact (HNSC)	10.93 (12.83)	0.66	15.87 (14.87)	0.79	8.57 (22.74)	0.67
Sexuality (HNSX)	22.67 (28.72)	0.93	37.33 (34.92)	0.95	24.83 (28.28)	0.95
Teeth (HNTE)	22.00 (32.72)	-	14.67 (26.22)	-	12.24 (26.08)	-
Problems opening mouth (HNOM)	10.67 (26.46)	-	14.00 (25.28)	-	10.20 (22.78)	-
Dry mouth (HNDR)	20.00 (30.86)	-	26.67 (22.34)	-	25.17 (23.10)	-
Sticky saliva (HNSS)	21.33 (33.51)	-	32.00 (26.90)	-	27.89 (26.66)	-
Coughing (HNCO)	26.00 (31.79)	-	20.00 (29.35)	-	19.73 (26.28)	-
Felt ill (HNFI)	26.00 (28.80)	-	22.67 (28.12)	-	13.61 (21.43)	-
Pain Killers (HNPK)	50.00 (50.51)	-	70.00 (46.29)	-	42.86 (50.00)	-
Nutritional supplements (HNNU)	14.00 (35.05)	-	12.00 (32.83)	-	6.12 (24.22)	-
Feeding tube (HNFE)	0.0 (00.00)	-	10.00 (30.33)	-	0.0 (00.00)	-
Weight gain (HNWG)	44.00 (50.14)	-	66.00 (47.85)	-	34.69 (48.09)	-
Weight gain (HNWG)	10.00 (30.33)	-	4.00 (19.79)	-	28.57 (45.64)	-

EORTC indicates European Organization for Research and Treatment of Cancer; ellipses, no coefficient α calculated (single-item scale).

## Results

The collected data related to sociodemographic characteristics and clinical variables of interest are shown in Table 1. The collected data related to quality of life, physiological functions, and symptoms in the evaluation periods are shown in Table 2 (EORTC QLQ-C30) and Table 3 (EORCT QLQ-H and N35). Both Tables contain their respective standard deviation (SD) and α coefficient.

*EORTC QLQ-C30 Questionnaires (* Figure 1 *)*

The comparison of locations (oral or oropharyngeal) in the assessment periods, showed significant statistical difference only in the period 1 week after treatment, where results were better in the oral group in emotional functioning (EF) (p=.001), cognitive functioning (CF) (p=.006), pain (PA) (p=.011), and appetite loss (AP) (p=.014). The comparison of treatment modalities (surgery or surgery plus radiotherapy) in the assessment periods, showed better results in the surgery group in the period before treatment for physical functioning (PF2) (p=.007), role functioning (RF2) (p=.010), pain (PA) (p=.018), appetite loss (AP) (p=.018), and constipation (CO) (p=.038). In the period 1 week after treatment, the results were better in the surgery group in cognitive functioning (CF) (p=.012), and in the surgery plus radiotherapy group in social functioning (SF) (p=.020) and financial difficulties (FI) (p=.041). In the period 3 months after treatment, the results were better in the surgery group in cognitive functioning (CF) (p=.044) and pain (PA) (p=.043).

The comparison of locations (oral compared to oral and oropharyngeal compared to oropharyngeal) in the assessment periods, showed improvement in the oral group in role functioning (RF2) (p=.004), fatigue (FA) (p=.022), and appetite loss (AP) (p=.018) between the periods before and 3 months after treatment. In the oropharyngeal group, there was improvement for role function (RF2) (p=.018), emotional function (EF) (p=.033), pain (PA) (p=.025), and appetite loss (AP) (p=0.010) between the periods of 1 week and 3 months after treatment, and there was a worsening in fatigue (FA) (p=0.011) between the periods before and 1 week after treatment.

The comparison of treatment modalities (surgery compared to surgery and surgery plus radiotherapy compared to surgery plus radiotherapy) in the assessment periods, showed a worsening in the surgery group in role functioning (RF2) (p=0.005), physical functioning (PF2) (p=.008), fatigue (FA) (p=0.044), and appetite loss (AP) (p=0.004) between the period before and 1 week after treatment. An improvement was verified between 1 week and 3 months after treatment in role functioning (RF2) (p=0.001), fatigue (FA) (p=0.008), and pain (PA) (p=0.045). In the surgery plus radiotherapy group, there was an improvement in the role functioning (RF2) between the periods 1 week and 3 months after treatment (p=0.047) only.

*EORCT QLQ-HandN35 Questionnaire (* Figure 2 *)*

The comparison of locations (oral or oropharyngeal) in the assessment periods, showed statistical difference only in the period 1 week after treatment with speech problems (HNSP) (p=0.019) and feeling ill (HNFI) (p= 0.027), with both results being better in the oral group. The comparison of treatment modalities (surgery or surgery plus radiotherapy) in the assessment periods, showed better results in the surgery group in swallowing (HNSW) (p<0.0001), speech problems (HSNP) (p=0.022), sexuality (HNSX) (p=0.006), and pain killers (HNPK) (p=0.012) in the period before treatment. In the period 1 week after treatment, there were better results in the surgery group in weight gain (HNWG) (p=0.021).

The comparison of locations (oral compared to oral and oropharyngeal compared to oropharyngeal) in the assessment periods, showed improvement in social contact (HSNC) (p=0.041) and weight gain (HNWG) (p=0.025) between 1 week and 3 months after treatment in the oral group. In the oropharyngeal group, there was an improvement in feeling ill (HNFI) (p=0.042) between 1 week and 3 months after treatment. There was a worsening in social eating (HNSO) (p=0.036) between the periods before and 1 week after treatment and in weight gain (HNWG) (p=0.025) between the periods 1 week and 3 months after treatment.

The comparison of treatment modalities (surgery compared to surgery and surgery plus radiotherapy compared to surgery plus radiotherapy), showed statistical difference only for the surgery group, where there was an improvement in pain (HPNA) (p=0.025) and trouble with social contact (HNSC) (p=0.008) between the periods of 1 week and 3 months after treatment. There was a worsening in the swallowing measurement (HNSW) (p<0.001) between before and 1 week after treatment, as well as in the periods before and 3 months after treatment (p=0.005). There was a worsening in the sexuality measurement (HNSX) (p=0.001) between the periods before and 1 week after treatment and in weight gain measurement (HNWG) between the periods before and 1 week after treatment (p=0.040) and between the periods 1 week and 3 months after treatment (p=0.005).

## Discussion

The null hypothesis was rejected because some quality of life and patient satisfaction scores varied according to the tumor location and treatment performed in the different assessment periods. 

A majority of males (82%) in this study, with a mean age of 61.5 years, were found to have a prevalence of SCC. Johnson et al., (2000) affirm that SCC corresponds to 90% of neoplasms found in the head and neck region and that there is a propensity of head and neck cancer for males, affecting mainly individuals over 50. They report that among the malignant tumor locations in men, the oral cavity is the ninth most common worldwide (Johnson et al., 2000). The individual profile of the patient is important to the quality of life, once sociodemographic factors can worsen or improve patient quality of life, and that social, economic, and cultural status have direct influence on recovery of the individuals affected by cancer (Babin et al., 2008).

There are several scales to assess the quality of life of people living with cancer, and they may focus on global health aspects, be specific for cancer, or be restricted to a type of neoplasm or treatment stage, measuring several aspects related to the physical and emotional health of the patient (Jaconbsen and Jim, 2011). Ojo et al., (2012), in their wide systematic review from 1990 to 2010, evaluated the use of instruments for assessment of the quality of life of patients with head and neck cancers, and found 57 different instruments for assessments. Jacobsen and Jim (2011) argue that the diversity of questionnaires for patients with cancer can be positive due to the possibility of different measurements, but can be negative because it complicates the comparison among different studies.

The graphical representation of EORTC QLQ-C30 results (Figure 1) shows that when comparing tumor locations, there were significant differences at only 1 week after treatment, which showed a more optimistic score in the oral group. The difference related to tumor localization can be explained by the functional complexity of the oral cavity that work in different functions such as speech, taste, and swallowing (Chandue et al., 2006). The significant difference of the quality of life scores in the period 1 week after treatment corroborates with some authors who explain that a decrease of these scores generally occurs immediately after the performed treatment. However, there is a tendency for these scores to return to their initial values after one year (So et al., 2012; Babin et al., 2008). It was verified that at the last assessment in this study, only three months after treatment, the results were close to initial values for many assessed criteria. 

When comparing the treatment modalities, most of the statistically significant results pointed towards worse quality of life scores for the irradiated patients. These results agree with studies that show patients treated by surgery with radiotherapy tend to show worse quality of life results (Rogers et al., 2006; Taylor et al., 2002). O’Neill et al., (2011) also affirm that quality of life results reported by patients are worse when given a greater dose of irradiation. This finding can be explained by the unpleasant side effects of radiotherapy on the head and neck area that can be experienced by some patients, which can include a decrease in mobility of superior members, a dry mouth feeling, a decrease of salivary flow and mucous saliva, a loss of gustatory sensitivity, swallowing difficulties, trismus, nausea, lymphedema, dental caries, mouth ulcers, infection, osteoradionecrosis, as well as skin problems and fatigue, among others (Taylor et al., 2002; American Society of Clinical Oncology, 2017).

On the other hand, when the comparison of groups was made individually in the different treatment periods, the majority of the worse quality of life results were found in the period 1 week after treatment. This fact denotes the psychological impact and sensitivity of the patient after treatment (Ojo et al., 2012). Among the scores that showed significant differences, pain (PA) and fatigue (FA) can be highlighted, since published literature states that pain is incapacitating, closely related to quality of life, and can influence physical functions and increase fatigue (Taylor et al., 2004). These two variables appear in all comparisons, except in the surgery plus radiotherapy group when compared to itself. 

The QLQ-H and N35 questionnaire results showed that the data were similar after 3 months, demonstrating no statistical difference when comparing the treatment modalities or the neoplasm locations. These data show that the quality of life rates were similar for patients in both the surgery and surgery plus radiotherapy groups after 3 months. The decrease in quality of life scores immediately after treatment, and their tendency to return to their initial values one year after the treatment (So et al., 2012; Babin et al., 2008), were observed in the present study. This showed that even after only 3 months, there is significant improvement in the quality of life, obtaining values close to the initial values.

The greater morbidity, which was observed at 1 week after treatment, can be caused by side effects that tend to disappear over time (Haj et al., 2016), which denotes the physical impact of the treatment, and the psychological impact that the diagnosis of cancer is capable of causing (Ojo et al., 2012), which emphasizes the importance of the short-term follow up. 

Although a significant difference in the scores may not necessarily be related to a clinically observed difference (Aaronson, 1993), it is important that the professional explores, together with the patient and a multidisciplinary team, the possibilities of treatment side effects and the importance and necessity of a recovery period after treatment that offers the patient a humanized clinical approach.

Limitations of this study were the absence of comparison between different disease stages, which can influence the quality of life and recovery capacity of patients being treated (Taylor et al., 2004; Allal et al., 2003), and the subjective answers given by patients to some questions of the questionnaires. Thus, future studies that explore quality of life shortly after oral/oropharyngeal cancer treatment by comparison of different disease stages could add better understanding about the impact of cancer treatment on health-related quality of life. The finding of this study can help the professional to understand how the treatment can impacts the quality of life and helps them to guide the patients about what they might expect to feel regarding the quality of life along the recovering period. 

In conclusion, quality of life is one of the factors that affects patients with SCC in the head and neck region. It is directly influenced by the time elapsed after treatment, whether with surgery or surgery plus radiotherapy. The period of greatest morbidity was 1 week after treatment, regardless of the modality performed, but scores close to the initial values were reestablished after 3 months.
